# Supplementary material for: Copy Number Variation of the PIGY Gene in Sheep and Its Association Analysis with Growth Traits
Source: Animals (Basel). 2020 Apr 15;10(4):688. doi: 10.3390/ani10040688 (PMC7222781; doi:10.3390/ani10040688)
Supplement: Supplementary file 1 [file animals-10-00688-s001.pdf]

animals

Article

Supplementary Material: Copy Number Variation of the *PIGY* Gene in Sheep and Its Association Analysis with Growth Traits

Ziting Feng [1], Xinyu Li [1], Jie Cheng [1], Rui Jiang [1], Ruolan Huang [1], Dingchuan Wang [1], Yongzhen Huang [1], Li Pi [2], Linyong Hu [2] and Hong Chen *

Table S1. The minimum value, mean value (SD) and maximum value of body size traits in three sheep.

Breeds	Body Size Traits	Min Value	Mean Value (SD)	Max Value
CKS	body weight (kg)	29.10	58.05 (SD = 13.24)	101.53
	body height (cm)	52	65.99 (SD = 3.69)	85
	body length (cm)	50	72.00 (SD = 6.87)	92
	chest circumference (cm)	67	81.62 (SD = 8.45)	111
HS	body weight (kg)	21.7	32.29 (SD = 4.54)	46.6
	body length (cm)	62	70.87 (SD = 3.85)	81
	chest circumference (cm)	65	76.76 (SD = 4.49)	90
	tube circumference (cm)	5	7.11 (SD = 0.55)	8.5
	body height (cm)	54.5	61.81 (SD = 3.71)	72
	rump width (cm)	14	17.44 (SD = 1.14)	20
STHS	chest deep (cm)	22	27.63 (SD = 2.62)	37.5
	tube circumference (cm)	5	7.17 (SD = 0.74)	8.8
	height at hip cross (cm)	53	62.89 (SD = 4.26)	74
	chest width (cm)	12.8	19.50 (SD = 3.42)	29
	chest circumference (cm)	59	72.08 (SD = 5.99)	91
	body height (cm)	51.5	63.28 (SD = 4.24)	74
	body length (cm)	40.5	59.05 (SD = 6.24)	74

© 2020 by the authors. Licensee MDPI, Basel, Switzerland. This article is an open access article distributed under the terms and conditions of the Creative Commons Attribution (CC BY) license (http://creativecommons.org/licenses/by/4.0/).
